# Extracellular microbes are required for mosquito development even in the presence of *Wolbachia*

**DOI:** 10.1371/journal.pntd.0013481

**Published:** 2025-09-05

**Authors:** Javier Serrato-Salas, Yanouk Epelboin, Danai Bemplidaki, Ivan Roger, Mathilde Gendrin

**Affiliations:** 1 Microbiota of Insect Vectors Group, Institut Pasteur de la Guyane, Cayenne, French Guiana; 2 Santé des Populations en Amazonie, lnserm, Cayenne, French Guiana; Connecticut Agricultural Experiment Station, UNITED STATES OF AMERICA

## Abstract

*Wolbachia,* an endosymbiotic bacterium infecting a wide array of invertebrates, has gained attention for its potential in vector control. Its capacity to colonise host populations primarily relies on vertical transmission and reproductive manipulation in arthropods. This endosymbiont is additionally mutualistic in some hosts, across several *Wolbachia* supergroups; notably, in nematodes and, as recently demonstrated, in planthoppers and bedbugs, it functions as an essential nutritional symbiont by providing vitamins to its host. Since mosquito larvae require microbe-derived nutrients for development, we investigated whether *Wolbachia* alone can support larval development in *Culex quinquefasciatus* mosquitoes. Our findings reveal that *Wolbachia* alone is insufficient to support larval development. Using transient colonisation with *Escherichia coli*, we developed a protocol to produce adult *Culex quinquefasciatus* mosquitoes harbouring *Wolbachia* only (germ-free^Wol+^). These results suggest that *E. coli* can support larval development in this species, which typically thrives in murky water; they also underscore the importance of extracellular microbes in larval growth. Furthermore, when *Wolbachia* infection was suppressed in germ-free^Wol+^ larvae using tetracycline treatment, we observed enhanced larval development, suggesting that *Wolbachia* acts as a metabolic parasite. In summary, this study opens the way for gnotobiology research in *Culex quinquefasciatus* and highlights the intricate interactions between *Wolbachia* and other members, which collectively influence mosquito development.

## Author summary

*Wolbachia* is a bacterium that infects many invertebrates and gained attention for its potential to curb transmission of dengue virus by mosquitoes. In some insect species, *Wolbachia* provides vitamins to its host. As gut bacteria provide essential vitamins for mosquito development, we investigated whether *Wolbachia* also fulfils this role. *Culex quinquefasciatus* species is highly colonised with *Wolbachia*, but its germ-free rearing was problematic so far. We developed a method to produce *Cx. quinquefasciatus* devoid of extracellular microbiota and demonstrated that *Wolbachia* alone cannot support larval development. However, we were able to produce adults harbouring *Wolbachia* only via transient colonisation of larvae with extracellular bacteria. The proportion of larvae reaching adulthood increased after tetracycline treatment against *Wolbachia*. Together, our data indicate that *Cx. quinquefasciatus* larvae, generally found in murky water, can grow in microbe-controlled conditions and suggest that *Wolbachia* acts as a metabolic burden in larvae rather than as a symbiont.

## Introduction

Mosquito-borne diseases pose a significant global health threat. Mosquitoes act as vectors of numerous medically significant pathogens, including arboviruses, nematodes that cause lymphatic filariasis, and protists that cause malaria. This highlights the urgent need for new vector control tools that are both safe for people and environmentally sustainable [1–6]. In insects, extracellular and intracellular microbes contribute essential microelements beyond the host genome’s metabolic capacities [7,8]. Mosquitoes experience distinct aquatic and terrestrial habitats during their life cycle, exposing them to a broad diversity of bacteria. The microbiota plays various roles, including larval nutritional support and direct impact on adult fitness, measured by lifespan and reproduction [9–15]. Mosquito larval development is more specifically dependent on B vitamins provided by their microbiota, in such a way that larval development is blocked in germ-free conditions and rescued by the addition of bacteria [8,9]. Like many insects, mosquitoes lack the complete metabolic pathways needed to synthesise B vitamins and rely on external sources for these essential nutrients [7,13,15,16]. Bacteria also support lipid, protein and nucleic acid metabolism in larvae [8,15,16].

*Wolbachia* is a genus of intracellular Alphaproteobacteria that infects many invertebrate species. It has attracted particular interest due to its diverse effects on hosts. In arthropods, *Wolbachia* is present in approximately 65% of terrestrial species and half of aquatic species [17,18]. Its genomic capacity for nutrient provisioning varies across strains. Many insect-associated strains biosynthesise riboflavin (B2), while the full biotin (B7) pathway has only been reported in bedbugs and two planthoppers strains. Partial pathways for thiamine (B1), pyridoxine (B6), and folate (B9) exist in most hosts [19]. For instance, *w*Mel enhances pyrimidine availability in *Drosophila*, accelerating larval development [20]. In filarial nematodes, *Wolbachia* provides haem for energy metabolism and enzyme function [19,21].

While *Wolbachia* remains a symbiont across host taxa, its interaction type varies: it acts as a nutritional mutualist in nematodes by supporting host metabolism, whereas in arthropods, it often functions as a reproductive parasite. This role involves manipulating host reproduction for its own benefit despite potentially imposing metabolic costs [19,21,22]. Within arthropods, *Wolbachia* is highly concentrated in female germlines, enabling vertical transmission while simultaneously acquiring resources for survival and growth [19]. Recently, species belonging to two hemipteran suborders have been found to maintain an unexpected nutritional link with *Wolbachia.* In bedbugs, *w*Cle (*Wolbachia* of *Cimex lectularius*) promotes nymph development, lifespan and fecundity by producing riboflavin and biotin [19,23,24]. In the planthopper species *Nilaparvata lugens* and *Laodelphax striatellus*, *Wolbachia*-cured insects are sterile and experimental reinfection with their *Wolbachia* (*w*Lug and *w*Stri, respectively) restores their fecundity levels [25].

Amongst dipterans, natural infections of *Wolbachia* have been found in some mosquito and fly species [26–28]. This includes common domestic mosquitoes like *Culex quinquefasciatus* and *Culex pipiens,* where *Wolbachia* is highly prevalent [29], in contrast to other mosquito species such as *Aedes aegypti* and most *Anopheles* species where *Wolbachia* has rarely been detected [30–32]. In mosquitoes, *Wolbachia* clearance in *Culex* mosquitoes delays egg laying and decreased lifespan and resistance to entomopathogenic bacteria, despite an increase in overall reproductive fitness [33,34]. *Wolbachia* also increases resistance to viral infection, via reducing RNA virus replication. When introduced in naturally *Wolbachia*-free species, some strains of *Wolbachia* can cut down transmission of dengue, chikungunya, and West Nile viruses [1–6,35–37]. In several countries, *Wolbachia*-transinfected *Ae. aegypti* have notably been released to colonise local populations with *Wolbachia*, rendering them poor arboviral vectors. This method has achieved significant progress in reducing dengue incidence in high burden settings worldwide [38,39].

So far, the role of *Wolbachia* in mosquitoes has been studied by antibiotic clearance of *Wolbachia* in conventionally-reared mosquito lines. This approach would not allow to detect potentially redundant roles between *Wolbachia* and extracellular bacteria. In particular, the high prevalence of *Wolbachia* in *Cx. quinquefasciatus* mosquitoes and the presence of a complete riboflavin-biosynthesis pathway in *w*Pip raise questions about its potential standalone role in host fitness.

Our laboratory recently developed a method to produce germ-free *Ae. aegypti* mosquitoes [13]. It relies on a transient colonisation of larvae with bacteria to support development; as we use an auxotrophic bacterial strain, it can be cleared by a change in diet to obtain germ-free adults. We decided to use this approach to assess the role of *Wolbachia* during development of *Cx. quinquefasciatus*, in otherwise germ-free or monocolonised conditions.

## Results

### Development of an egg sterilisation protocol for *Culex* quinquefasciatus

Mosquito production in sterile conditions requires protocol optimisation, considering the specificities of mosquito species. While gnotobiotic *Ae. aegypti* (i.e. carrying a defined microbiota composition) have now been reared in several laboratories, we needed to define conditions to rear *Culex* mosquitoes in microbiologically-controlled conditions. To this aim, we used as a starting point a transient colonisation method that was previously developed for *Ae. aegypti* in our laboratory; larval development is supported via monocolonisation with a bacterium that is lost at the time of metamorphosis [13]. This consists of a three-step procedure. First, eggs are surface-sterilised, producing germ-free larvae. Second, newly hatched germ-free larvae are reared in the presence of a mutant bacterium, *E. coli* HA416 (AUX), which is auxotrophic for two amino-acids essential for peptidoglycan biosynthesis. As long as larvae are provided with a special sterile diet supplemented with these amino acids, AUX grows and supports larval development. Third, pupae are transferred to a rearing environment devoid of the specific amino acids, allowing the emergence of germ-free adults. We decided to adapt this approach for field-collected *Cx. quinquefasciatus* egg rafts to produce mosquitoes with no culturable microbiota*.*

**Fig 1:**
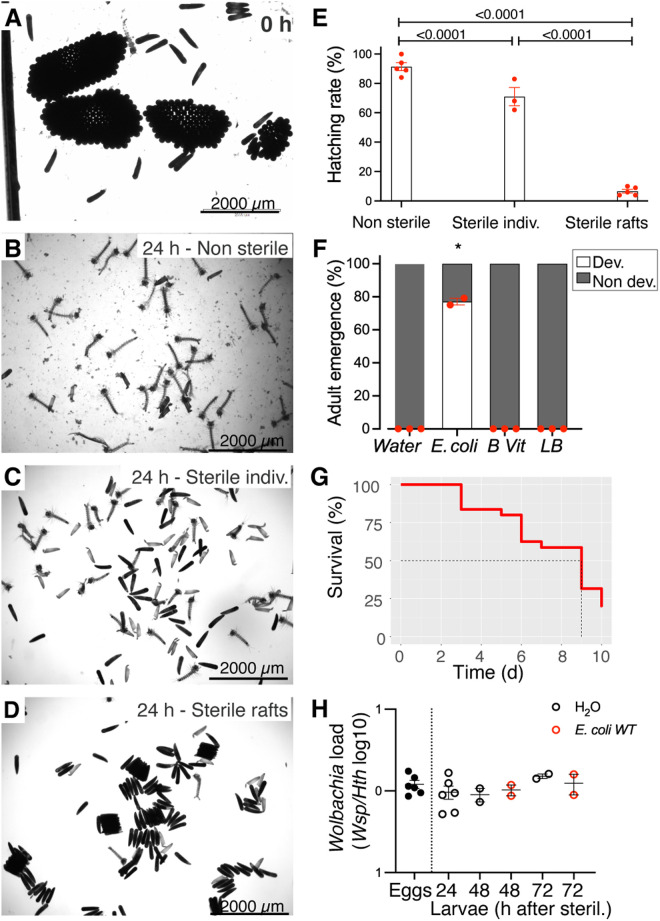
*Culex quinquefasciatus* germ-free*^Wol^*^+^ larvae hatch but do not develop without extracellular bacteria. **A.** *Cx. quinquefasciatus* egg rafts collected from a local breeding site (0 h post-collection). **B-D.** Flask content 24 h after treatment: (**B**) larvae eclosed from non-sterile untreated rafts; (**C**) sterile individuals, i.e. after sterilisation and harsh fragmentation of rafts into single eggs; (**D**) outcome of sterile rafts, whose structural integrity was preserved over sterilisation. **E**. Hatching rates for non-sterile, sterile individuals and sterile rafts groups. **F**. Larval development success of larvae under sterile diets: Sterile water (H_2_O), bacterial culture (*E. coli* WT), B vitamins solution (B vit) and undiluted LB medium (LB). Sterile diet vs *E. coli* WT (p < 0.0001). **G**. Survival curve of sterile larvae fed autoclaved baby fish food. **H**. *Wolbachia* load in newly hatched sterilised larvae with the influence of bacterial culture. **Abbreviations:** Sterile indiv. (Sterile individuals), B vits (B vitamin cocktail), LB (lysogeny broth). **Statistical analysis: E**: Generalised Linear Mixed Models (GLMMs) was used with binomial distribution and Bonferroni adjustment; **F, H**: Linear Mixed Models were applied (Type III ANOVA with Satterthwaite´s method). **Replicates: A-D**: Representative images from 3 independent biological replicates. **E**: Mean ± SEM (5 independent biological replicates and 3 replicates for sterile indiv. group). **F**: Mean ± SEM (3 independent biological replicates). **G**: Mean ± 95% confidence interval (4 independent biological replicates). **H**: Mean ± SEM (2 independent biological replicates, each based on several mosquito pools). Individual sample sizes and statistical summaries are provided in S1 Table.

*Ae. aegypti* eggs are laid individually and survive for weeks after drying; their hatching is stimulated when they are covered with water, notably due to the lack of oxygen. In contrast, *Cx. quinquefasciatus* eggs are laid as rafts containing up to several dozens of eggs (Fig 1A), which float on the water surface. These eggs remain wet and close to the water surface throughout their uninterrupted development, limiting laboratory flexibility. When using the *Ae. aegypti* egg-sterilisation protocol to surface-sterilise *Cx. quinquefasciatus* eggs, we observed that the egg rafts disaggregated into individual eggs or small clusters, sinking to the bottom of the flask placed in vertical position, filled 2/3 with sterile water. Almost no larvae hatched in these conditions.

*Culex* egg rafts are naturally buoyant and float on water surface, getting direct access to atmospheric oxygen through the raft’s hydrophobic outer layer [40]. We hypothesised that the harsh procedure likely disrupts the raft’s structural integrity, causing eggs to sink or exposing submerged eggs to hypoxia. We thus decided to perform a delicate handling, minimising raft disintegration when submerging it in sterilising solutions (sodium hypochlorite and ethanol). In parallel, we fully disintegrated another subset of rafts, so that all the eggs are separated. To keep eggs close to water surface for gas exchange, we incubated eggs in a minimal water layer, 1–2 mm deep (3–5 mL in a 25 cm^2^ flask laid horizontally). The next day, only 6.6% of preserved sterilised rafts hatched, compared to 71% of individualised sterile eggs (Fig 1B - 1E). Non-sterile eggs exhibited the highest hatching rate (91%).

The presence of *Wolbachia* in sterile larvae was confirmed by qPCR, and egg sterility was verified after incubation in liquid Lysogeny Broth medium (LB) or on LB agar plates. We thus refer to these individuals as germ-free^*Wol+*^, indicating they are germ-free except for the presence of the *Wolbachia* endosymbiont. To assess whether *Wolbachia* alone could support larval development, we monitored the development and lifespan of larvae provided sterile conventional food for 10 days. Larvae remained in their first instar, and supplementing diet with a cocktail of B vitamins that are essential for *Cx. quinquefasciatus* larval development [41] or with medium for bacterial culture did not improve development (Fig 1F). However, these larvae were still able to develop, as provision of live bacteria efficiently rescued 77% of individuals to adulthood. Approximately 80% of sterile larvae survived until day 5, but survival sharply declined in the following days, resulting in around 20% of surviving individuals on day 10 (Fig 1G). We confirmed by qPCR that *Wolbachia* colonisation was unaffected by the absence of other bacteria. *Wolbachia* levels remained stable in first instar larvae even after 72 h, independently of the presence of *E. coli* (Fig 1H).

Together, these data indicate that *Wolbachia* alone is insufficient to support larval development in the absence of other bacteria. Furthermore, although *Cx. quinquefasciatus* typically thrives in a microbe-rich environment compared to other mosquito larvae, the sole presence of *E. coli* is sufficient to complement its requirements for larval development.

### Production of germ-free*^Wol^*^+^ *Culex* quinquefasciatus adults

**Fig 2:**
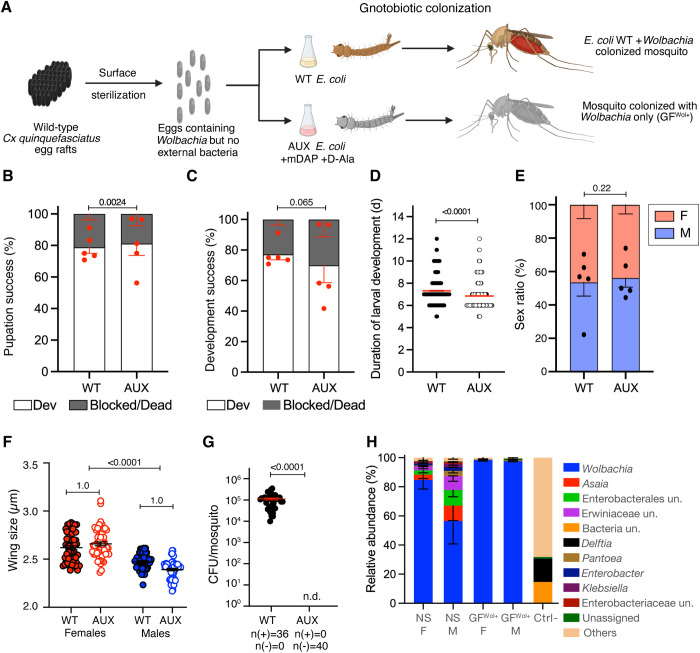
Transient colonisation enables production of germ-free^*Wol*^^+^ adults. **A.** Experimental workflow for rearing germ-free^*Wol*+^ *or* monocolonised larvae (created in https://biorender.com). **B.** Pupation success rates (% of 1^st^ instar larvae reaching pupal stage). **C.** Developmental success to adulthood (% of 1^st^ instar larvae reaching adulthood). **D.** Larval development duration to pupae. **E.** Proportion of males and females among adults. Sex was assessed immediately after adult emergence. **F.** Wing size (read out of adult size). **G.** Bacterial load (CFU/mosquito) 10 days after emergence: AUX vs WT. **H.** Bacterial relative abundance in mosquito samples. Bar plot shows the proportion of the most prevalent bacterial taxa detected in each experimental group: non ster. - F (non-sterile females), non ster.-M (non-sterile males), GF^*Wol*+^-F (germ-free*^Wol^*^+^ females), GF^*Wol*+^-M (germ-free^*Wol*+^ males), and mock (negative controls of DNA extraction). Taxa are grouped by genus or higher taxonomic level as indicated on the x-axis. “Unassigned” (and “un.”) denotes sequences not classified at the indicated taxonomic rank. “Others” includes all remaining taxa with <0.4% reads. **Abbreviations:** WT (individuals colonised with wild-type *E. coli*)*,* AUX (individuals colonised with auxotrophic *E. coli* HA416). n.d.: non detected. n(+): number of individuals harbouring 1 or more CFUs. n(-): number of individuals harbouring 0 CFU. *Un. – Unassigned.* **Statistical analysis: B**, **C**, **E**: Generalised Linear Mixed Models were used with binomial distribution. **D**, **F** and **G**: Linear Mixed Models were applied (Type III ANOVA using Satterthwaite´s method). **Replicates: B**-**F**: Mean ± SEM (5 independent biological replicates), **G-H**: Mean ± SEM (3 independent biological replicates). Individual sample sizes and statistical summaries are provided in S1 Table.

Having successfully produced germ-free^*Wol*+^ larvae, we investigated whether the transient colonisation approach could yield germ-free^*Wol*+^ adults. To accomplish this, we added the auxotrophic bacterial culture (*E. coli* HA416) alongside a wild-type *E. coli* (WT) as a positive control (Fig 2A).

**Fig 3:**
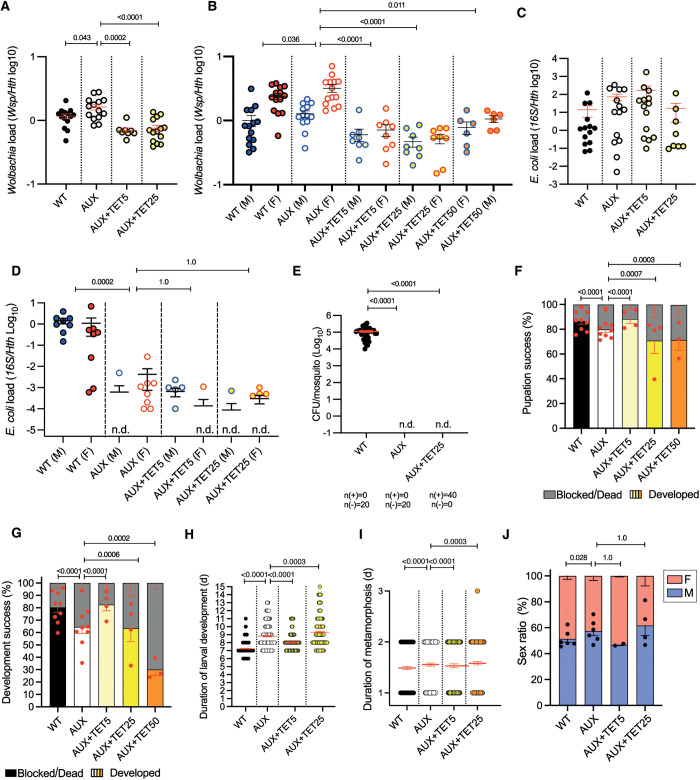
*Wolbachia* negatively impacts development success. **A-B.** qPCR quantification of *Wolbachia wsp* DNA in larvae (**A**) and in adults (**B**). **C-D.** qPCR quantification of *E. coli* 16S gene in larvae (**C**) and adults (**D**). No significant differences were found in larvae. **E**. Bacterial decolonisation measured by CFU assay in adults. **F**. Pupation rate (% of initial 1^st^-instar larvae reaching pupal stage). **G**. Adult emergence rate (% of initial 1^st^-instar larvae reaching adulthood). **H-I.** Larval development timings to pupae (**H**) and to adult emergence (**I**). J. Proportion of males and females among adults. **Abbreviations:** WT (individuals colonised with wild-type *E. coli*), AUX (individuals colonised with auxotrophic *E. coli*), TET (tetracycline concentration applied in µg/mL). n(+): number of individuals harbouring 1 or more CFUs. n(-): number of individuals harbouring 0 CFU. **Statistical analysis:** A-E, H, I: Linear Mixed Models were applied with Satterthwaite´s method. F-G, J: Generalised Linear Mixed Models were used with Bonferroni´s adjustment. **Replicates:** A-E: Mean ± SEM (3 independent biological replicates), F-H: Mean ± SEM (3-9 independent biological replicates: 9 for WT and AUX, 4 for TET5 and TET25, 3 for TET50). J: Mean ± SEM (2-6 independent biological replicates: 6 for WT and AUX, 4 for TET25, 2 for TET5). Individual sample sizes and statistical summaries are provided in S1 Table.

We monitored development daily and found minor differences in development success rates or timing between larvae monocolonised with WT *E. coli* and those reared with the auxotrophic strain (AUX). In the AUX group, larval development success to pupa was slightly higher, while overall development to adulthood was marginally significantly lower (Figs 2B - 2E and S1 Fig). Sex ratios and adult wing size, used as a proxy of the adult size, were also comparable between groups (Fig 2F - 2G). Re-analysis of developmental rates across subsequent experiments presented in Fig 3 revealed that the AUX group exhibited an overall lower development rate to adulthood and a significantly longer larval-to-pupal development time (pupae: p = 0.007, adults: p < 0.0001) based on a generalised linear mixed model (GLMM) with least square means, though replicate variability partially masked these trends when considering Fig 2 only. Pupal transfers were conducted under sterile conditions, and colony-forming unit (CFU) assays indicated that all 10-day old gnotobiotic mosquitoes reared with WT retained *E. coli* colonisation, whereas germ-free^*Wol*+^ mosquitoes did not (Fig 2H).

### Impact of *Wolbachia* on development success of germ-free *Culex* *quinquefasciatus* mosquitoes

*Wolbachia* alone is insufficient to support larval development in germ-free^*Wol*+^ *Cx. quinquefasciatus* mosquitoes. It may have a nutritional role in iron metabolism, biosynthesis of nucleotides and riboflavin (B2), but its genome lacks folate (B9) and biotin (B7) pathways. Given that bacterial-derived riboflavin and folate are essential for larval development, and *Wolbachia* provides purines/pyrimidines to fly larvae [13,15,24,42,43]. We tested whether *Wolbachia* could compensate for suboptimal *E. coli* nutrient provision. Alternatively, we hypothesised that *Wolbachia* might act as a metabolic burden by competing for nutrients. To investigate this, we reduced *Wolbachia* load in mono-colonised larvae using tetracycline, a widely used antibiotic to clear *Wolbachia* in insects, including *Culex* mosquitoes [44,45]. We took advantage of the fact that our auxotrophic *E. coli* strain (AUX), used for transient colonisation, carries a tetracycline-resistance cassette [46]. We treated AUX-colonised larvae with tetracycline (5, 25, or 50 µg/mL) to assess developmental outcomes [47]. All doses significantly reduced *Wolbachia* loads in larvae and adults (Fig 3A: AUX vs AUX + TET25 – p < 0.0001, AUX vs AUX + TET5 – p = 0.0002, AUX + TET25 vs AUX + TET5 – p = 1.0; 3B: AUX vs AUX + TET25 – p < 0.0001, AUX + TET25 – p < 0.0001, AUX vs AUX + TET50 – p = 0.011, AUX + TET25 vs AUX + TET5 – p = 1.0, AUX + TET25 vs AUX + TET50 – p = 1.0). Development success was concentration-dependent: 5 µg/mL tetracycline increased development success to adulthood, which would indicate that *Wolbachia* is a metabolic burden. The highest dose (50 µg/mL) however decreased success, and the intermediate concentration (25 µg/mL) led to an intermediate development success (Fig 3F and 3G).

AUX-colonised larvae exhibited delayed development compared to WT-colonised controls, partially rescued by 5 µg/mL tetracycline (Fig 3H and 3I). AUX conditions also produced fewer females (AUX vs WT – p = 0.028), a potential indicator of nutritional stress, as females require more bacteria-derived resources than males to develop [13,16] (Fig 3J). We confirmed by CFU assays the persistence of *E. coli* in WT-colonised mosquitoes but not in AUX or AUX + TET groups (Fig 3E), consistent with post-metamorphosis bacterial loss (Fig 3C and 3D).

We considered three hypotheses to interpret these contrasting impacts of different concentrations. Firstly, *Wolbachia* would impose a burden to larvae, and some larvae would die at higher concentrations due to tetracycline toxicity. Secondly, the increase at low antibiotic concentration may be due to hormesis, a stress response induced by a toxic compound at very low concentrations that can positively affect fitness. Thirdly, the impact of *Wolbachia* would be concentration-dependent effect of *Wolbachia*, where a low amount of bacteria may promote development while a too high *Wolbachia* load would impose a metabolic burden. As we did not detect any difference in the *Wolbachia* load between the three concentrations of tetracycline, we further investigated the bacteria-independent impact of tetracycline on mosquitoes, using *Ae. aegypti* as a *Wolbachia*-free model.

### Bacteria-independent effect of tetracycline on mosquito development

*Ae. aegypti* larvae of the New Orleans strain were treated with tetracycline at concentrations ranging from 5 to 200 μg/mL. To ensure that the observed effects were directly attributable to the antibiotic treatment rather than to vehicle effects, we also included mock controls with matching concentrations of ethanol.

**Fig 4:**
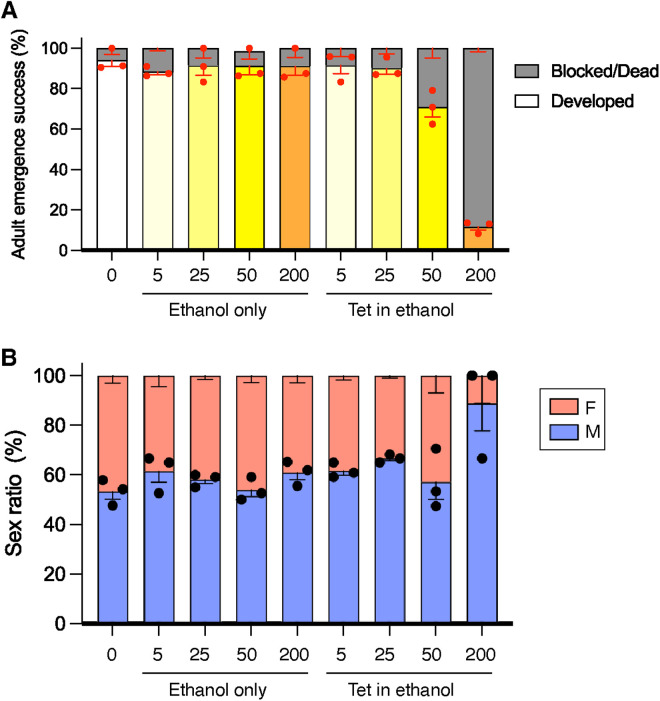
Tetracycline dose-response effects on larval development in *Aedes aegypti.* **A.** Adult emergence success showing proportion of larvae that successfully developed to adulthood versus those that died or were developmentally arrested. Data represent mean ± SEM from three independent biological replicates. *Ae. aegypti* New Orleans strain (*Wolbachia*-free) was used to isolate antibiotic effects from endosymbiont interactions. **B.** Sex ratio of emerged adults following larval tetracycline treatment. Statistical analysis: Generalised Linear Mixed Models with binomial distribution were applied to emergence success data. Significant differences were observed between TET200 and all other treatments (p < 1e-12), and between TET0 and TET50 (p = 0.037). No sex ratio differences were found in any other condition.

Adult emergence success showed clear dose-dependent antibiotic toxicity (Fig 4A). Low tetracycline concentrations (5–25 μg/mL) maintained high development success (90.0-91.5% adult emergence), comparable to vehicle controls (88.2-92.9%) and untreated larvae (94.1%). In contrast, higher concentrations demonstrated severe toxicity: 50 μg/mL tetracycline significantly reduced adult emergence to 70.8% (p = 0.037 vs control), while 200 μg/mL was highly lethal with only 11.6% successful development to adulthood. Ethanol vehicle controls showed no adverse effects across all tested concentrations, confirming that observed toxicity was antibiotic-specific rather than solvent-related.

Sex ratio remained stable across most treatments (Fig 4B). Both tetracycline treatments and ethanol vehicle controls at concentrations of 5–50 μg/mL maintained approximately balanced sex ratios (~50% females), with no significant deviations from controls. However, the highest tetracycline concentration (200 μg/mL) resulted in a pronounced male-biased sex ratio (87.5% males, 12.5% females), indicating preferential female mortality at this toxic dose.

These findings indicate that in *Ae. aegypti*, tetracycline doses ≥50 μg/mL exhibit direct larval toxicity. This points that the positive impact observed in *Cx. quinquefasciatus* at low tetracycline concentration are most likely attributable to antibiotic effects on bacterial symbionts rather than non-specific drug toxicity.

## Discussion

In this study, we established a protocol to produce *Culex* mosquitoes harbouring solely *Wolbachia* and no other microbiota. We demonstrated that *Wolbachia* alone is insufficient to support larval development. Conversely, our data indicate that this endosymbiont negatively impacts mosquito larval development.

While sterile rearing of *Aedes* species has been optimised over the past decade, enabling functional studies of their microbiota, producing gnotobiotic *Culex* has remained challenging. A recent study showed that *Ae. albopictus* recruits largely similar microbiomes as non*-Wolbachia* dominated species, suggesting *Wolbachia* presence does not influence microbiome assembly [9,10,13,48]. *Culex* mosquitoes typically breed in turbid, microbe-rich water [49–51], whereas *Aedes* prefer cleaner environments [52]. For example, *Aedes* oviposition traps use clear water, while *Culex* attraction often requires organic additives like chicken manure*.* Considering these differences, we were not sure that gnotobiotic *Cx. quinquefasciatus* larvae would grow in the absence of a complex microbial community. However, we found that *E. coli* alone rescues larval development in *Cx. quinquefasciatus,* albeit with slightly lower success (~80% with WT and ~65% with AUX compared to *Ae. aegypti* (~90%, Fig 4A and [13]). This suggests quantitative, rather than qualitative, differences in microbial metabolite requirements between the two species.

Another critical distinction lies in egg-laying behaviour: *Culex* eggs form rafts that float in water, keeping their upper surface dry and oxygenated. Our sterilisation protocol caused eggs to sink, resulting in low hatching rates unless individual eggs were placed in thin water layers. We attribute this to *Culex*’s high oxygen demand during embryogenesis [40,53,54].

*Wolbachia* prevalence in insects comes from reproduction manipulation (i.e., cytoplasmic incompatibility), vertical transmission efficiency, and host stability. Our observations are in line with the high colonisation capacity in *Cx. quinquefasciatus*, as symbiotic DNA is still detected even after tetracycline treatment at high concentrations. Complete eradication typically requires multi-generational antibiotic treatment [44].

Some *Wolbachia* strains combine reproductive parasitism and positive impact on other aspects of host fitness, and have as such been qualified as “Jekyll and Hyde” symbionts [55]. *Wolbachia* notably provides riboflavin and biotin to bedbugs [19,24] and planthoppers [25]. It also enhances *Drosophila* stress resistance by modulating energy metabolism [42]. Our data do not point to nutritional support of *Wolbachia* in *Cx. quinquefasciatus* larvae. Although *Culex*-associated *Wolbachia* (*w*Pip) encodes riboflavin biosynthesis pathway [25], its clearance improved larval development in our study, suggesting that it is a burden rather than a help for larval development. This aligns with a non-significant trend of reduced development success in *Wolbachia-*infected *Ae. albopictus* [56]. The highest antibiotic concentration likely inhibited development via tetracycline toxicity, as it does not clear *Wolbachia* more efficiently than lower concentration and as it also affects *Wolbachia*-free *Ae. aegypti* larvae. Further work based on the production of a *Wolbachia*-cleared line of *Cx. quinquefasciatus* would however allow to fully clarify the impact of *Wolbachia* on larval development. Whilst *Wolbachia* does not appear to provide benefit to mosquito metabolism, its antiviral protection effect in adult mosquitoes may be considered as a “Jekyll and Hyde” way to promote its evolutionary success [57,58].

Our findings demonstrate a concentration-dependent toxicity of tetracycline in larvae, establishing optimal working concentrations for gnotobiotic studies. While concentrations of 5–25 μg/mL maintained high development success (90-91.5% for *Ae. aegypti*) comparable to controls, doses ≥50 μg/mL exhibited significant toxicity: 50 μg/mL reduced adult emergence to 70.8% (p = 0.037 vs control), while 200 μg/mL was severely lethal with only 11.6% survival (p < 1e-12). The highest concentration also caused pronounced sex ratio distortion (87.5% males), indicating preferential female mortality. This may be due to direct toxicity or to a lack of nutrients, as females require more bacteria-derived nutrients than males for development [14]. Tetracycline targets bacterial 30S ribosomes, mitochondrial ribosomes in eukaryotes may be secondary targets [59]. Prior studies attributed tetracycline’s negative effects to microbiota loss [60], but conventional rearing approaches cannot distinguish between antibiotic toxicity and microbial depletion effects. Our gnotobiotic approach based on a tetracycline-resistant bacterium and transient colonisation, definitively demonstrates direct antibiotic toxicity independent of microbiota effects. This finding aligns with documented mitochondrial dysfunction in other systems, and explains developmental delays observed in various organisms following tetracycline exposure [61].

Tetracycline degradation products (anhydrotetracycline and epitetracycline) exacerbate toxicity in aquatic organisms [33,44,47,62]. We observed larval water browning over time, indicating oxidation. However, whether the observed toxicity stems from tetracycline or its by-products remains to be determined. Regardless, our dose-response data establish that concentrations ≤25 μg/mL are suitable for controlled microbiota studies while avoiding confounding toxicity effects.

## Conclusions

While studies investigating *Wolbachia*’s impact on mosquito fitness are often confounded by antibiotic-induced disruptions to the native microbiota, we developed a methodology to isolate its role in *Culex* mosquitoes devoid of other microbiota partners. Our findings demonstrate that *Wolbachia* cannot independently support larval development, consistent with the absence of a biotin biosynthesis operon in *Wolbachia* strain *wPip*, unlike bedbug- and planthoppers-associated strains. On the contrary, our data point to a parasitic effect of *Wolbachia* during larval development, underscoring that its reproductive manipulation strategies (i.e., cytoplasmic incompatibility) alone are sufficient to explain its widespread prevalence natural *Cx. quinquefasciatus* populations.

## Materials and Methods

### Culex quinquefasciatus mosquitoes

Up to three breeding sites per replicate were established at the *Institut Pasteur de la Guyane* in Cayenne, French Guiana (GPS: 4.943100, -52.325350). Each site consisted of containers filled with tap water, soil and chicken manure to attract wild gravid *Cx. quinquefasciatus* females. Sites were checked daily, covered with nets on weekends, and replaced every 14 days (or more frequently) to prevent mosquito emergence from uncollected larvae. Mosquito species identification was confirmed using taxonomic keys [63,64]. *Cx. quinquefasciatus* dominates urban areas in French Guiana [64], subsequent microscopic examination of egg rafts, larvae and adults revealed no morphological variations.

### Sterilisation protocol and bacterial inoculum

Egg rafts were collected early in the day, pooled from all breeding sites, transported to the laboratory and pre-washed with tap water to remove organic debris. In a biosafety cabinet, rafts were sterilised as previously described [13]. Sterile eggs were incubated in 25 cm^2^ culture flasks with a minimal water layer (3–5 mL) in horizontal position in a climate-controlled chamber at 28°C with a 12-hour light-dark cycle and 80% relative humidity for 24 h. Sterility was confirmed by plating egg samples on Lysogeny Broth (LB) agar for 48 h. *E. coli* HA416 (AUX) culture was prepared as previously described [13].

Newly hatched first-instar larvae were individually transferred to 24-well plates, each well containing 1.5 mL of *E. coli* HA416 culture (supplemented with 50 µg/mL D-Alanine and 12.5 µg/mL meso-diaminopimelic acid) and ~0.05 mL of sterile 5% (w/v) baby fish food solution. For each experiment, 3–5 *Culex* egg rafts were sterilised and the resulting two flasks were found to hatch around 1,000 sterile larvae each. Larvae were distributed into 24-well plates at a density of one larva per well, with at least 15 plates per independent biological replicate to ensure statistical power. Plates were maintained in a climate-controlled chamber at 28°C with a 12-hour light-dark cycle and 80% relative humidity. Wild-type *E. coli* HS (WT) was used as a control.

### Larval development monitoring and adult manipulation

Hatching rates were quantified microscopically 24 h post-sterilisation. Survival, pupation and development success were recorded twice daily for 14 days. After pupae transfer in sterile cups with cotton and sugar, sex was recorded by verifying the appearance of antenna fibrils, maxillary palps and abdominal apex.

Larvae were maintained under sterile conditions. Upon pupation, individuals were transferred to a sterilised plastic container for adult emergence. A small flat-bottom tube containing sterile cotton and 10% sterile sucrose solution was added to the container to provide nourishment for emerging adults. Adults were collected 3–5 days post emergence for wing size quantification. They were anesthetised on CO_2_, washed in 75% Ethanol. Wings were dissected and fixed in 4% paraformaldehyde and mounted on a microscope slide for observation at a 20x magnification.

### Assessment of bacterial decolonisation

To evaluate bacterial decolonisation, 10-day old adults were anesthetised with CO_2_ and processed in a biosafety cabinet. Male and female specimens were collected and homogenised with sterile water using sterile pestles and an electric tissue homogeniser to achieve a uniform suspension. Serial dilutions of the homogenate were prepared in sterile water and plated onto LB agar plates without antibiotics. Plates were placed in an incubator at 30°C, and colony-forming units were counted after 24 h.

### Antibiotic treatment during transient colonisation rearing

To assess the role of *Wolbachia*, larvae colonised with tetracycline-resistant *E. coli* HA416 (AUX) were treated with tetracycline (5, 25 and 50 μg/mL) to each larval well. Antibiotic doses were replenished on days 3 and 5, and bacterial cultures were supplemented on days 2 and 4 to maintain colonisation [44,45].

### *Wolbachia* detection

Individual mosquitoes were homogenised in lysis buffer using a Precellys Evolution bead beater homogeniser (Bertin Technologies). For sterile larvae, pools of ≥30 first-instar larvae were processed per sample. DNA was extracted with the MagBio HighPrep Blood and Tissue DNA kit on a KingFisher Duo Prime system (Thermo Scientific), following proteinase K digestion (55°C overnight). DNA concentration was adjusted to 25 ng/μL (Nanodrop spectrophotometer, Thermo Scientific), with DNA normalised post-extraction to ensure consistent input for qPCR. *Wolbachia* density was quantified via qPCR (Roche LightCycler 480) using *wsp* primers:

*wsp*-Fwd 5’-ATCTTTTATAGCTGGTGGTGGT-3’, *wsp*-Rev 5’ -AAAGTCCCTCAACATCAACCC -3’. Mosquito homothorax (*hth)* served as the endogenous control: *hth*-Fwd 5’- TGGTCCTATATTGGCGAGCTA – 3’, *hth*-Rev 5’-TCGTTTTTGCAAGAAGGTCA – 3’ [65]. Technical duplicates were performed for qPCR.

### Microbiome characterisation of germ-free^Wol+^ mosquitoes

Germ-free^Wol+^ mosquitoes and *E. coli* WT colonised-mosquitoes were reared as described previously. DNA samples were extracted included controls to monitor contamination. The V3-V4 hypervariable region of the 16S rRNA gene was amplified using primers 338F (5′-ACTCCTACGGGAGGCAGCA-3′) and 806R (5′-GGACTACHVGGGTWTCTAAT-3′). Libraries were prepared following the Illumina MiSeq platform guidelines, with paired-end sequencing (2 × 250 bp) performed to characterise bacterial communities. Raw sequences were imported into QIIME2 (v2024.10) using a file linking sample IDs to forward/reverse read paths. Primer sequences were trimmed with cutadapt, and reads were quality-filtered (Phred score ≥ 20). Denoising was performed with DADA2, truncating forward reads at 228 bp and reverse reads at 226 bp to remove low-quality regions. Chimeras were filtered out, yielding amplicon sequence variants (ASVs). ASVs were classified against the SILVA 138 database using a Naive Bayes classifier trained on primer-specific reference sequences.

### Statistical analyses

Data were analysed in RStudio (v2023.06.2 + 561; R v4.3.1) using survival curves (survival and survminer packages) and generalised linear mixed models (lme4, lmertest and emmeans packages). Figures were generated in GraphPad Prism (v10.3.1) [66,67]. For qPCR, technical duplicates were used. The term “replicate” otherwise refers to “biological replicate” throughout the manuscript.

## Supporting information

S1 Table Statistical details for each Figure item. (XLSX)

S1 Fig Duration of metamorphosis, i.e., duration between pupa appearance and adult emergence. Each dot represents one single individual. Stats: Linear Mixed Models were applied (Type III ANOVA using Satterthwaite´s method). Replicates: Mean ± SEM (5 independent biological replicates). Individual sample sizes and statistical summaries are provided in S1 Table.
(TIF)
