# Supplementary figures and images for: Extracellular microbes are required for mosquito development even in the presence of Wolbachia
Source: PLoS Negl Trop Dis. 2025 Sep 5;19(9):e0013481. doi: 10.1371/journal.pntd.0013481 (PMC13252830; doi:10.1371/journal.pntd.0013481)

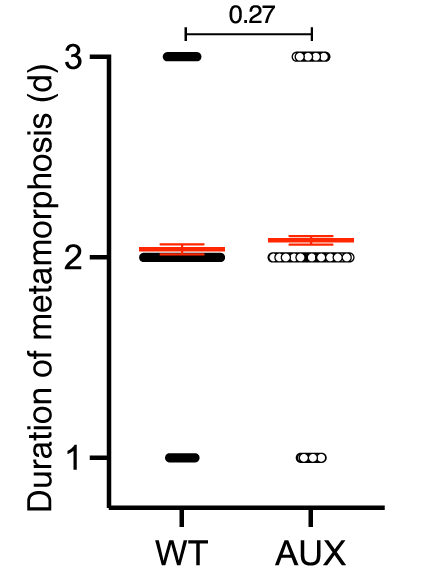

Supplement: S1 Fig — Each dot represents one single individual. Stats: Linear Mixed Models were applied (Type III ANOVA using Satterthwaite´s method). Replicates: Mean ± SEM (5 independent biological replicates). Individual sample sizes and statistical summaries are provided in S1 Table. (TIF) [file pntd.0013481.s002.tif]
