# Supplementary material for: Extracellular microbes are required for mosquito development even in the presence of Wolbachia
Source: PLoS Negl Trop Dis. 2025 Sep 5;19(9):e0013481. doi: 10.1371/journal.pntd.0013481 (PMC13252830; doi:10.1371/journal.pntd.0013481)
Supplement: Attachment — Submitted filename: Manuscript Number PNTD-D-25-00250.docx [file pntd.0013481.s003.docx]

Extracellular microbes are required for mosquito development even in the presence of *Wolbachia*

This study investigates the role of extracellular microbes in *Culex quinquefasciatus* larval development when *Wolbachia* is present. By creating a gnotobiotic rearing system, the researchers were able to generate *Culex* mosquitoes that have only *Wolbachia* (germ-freeWol+), allowing them to ascertain whether *Wolbachia* alone can support larval development. The study shows that *Wolbachia* alone is not enough for development and may even impose metabolic cost on larvae. These findings fit into a broader framework of *Wolbachia*-host interactions and the overall significance of extracellular microbiota in mosquito physiology. However, certain data presentation, interpretation of metabolic interactions, and figure legibility should be improved.

**Abstract:**	The abstract provides a concise overview of the study, explaining the role of *Wolbachia* in mosquito development and the significance of extracellular microbes in *Culex quinquefasciatus*. Certain aspects require clarification, and the conclusion of *Wolbachia* as a "metabolic parasite" is not adequately supported

- Line 14 - Clarify which insects rely on *Wolbachia* for nutrition to strengthen this claim
- Line 21 - Experiment shows that *E. coli* can support development but this does not mean *Culex* mosquitoes rely only on *E. coli* in natural conditions -- "which generally grows in dirty water" could be misinterpreted as suggesting that *E. coli* is the dominant bacterium in natural *Culex* habitats. I suggest -- *E. coli* can transiently support development in the absence of native microbiota, demonstrating the role of extracellular microbes in larval growth.
- Line 25 - this statement overgeneralizes the findings –I suggest …gnotobiology studies in *Culex quinquefasciatus* and highlight how *Wolbachia* interacts with other microbiota to influence mosquito development.

**Author summary:**

- Line 33: Culex quinquefasciatus – not italicised
- Line 40: Wolbachia - not italicised
- Line 41: incorrectly suggests that *Wolbachia* stops being a symbiont when it imposes a metabolic burden, which is not correct symbiont can be mutualistic, commensal, or parasitic.

**Introduction**

- Line 69: *Wolbachi*a's reproductive manipulations (CI, male-killing, feminization while relevant, does not directly relate to mosquito or mosquito development—needs to be removed –authours should concetrate instead summarizing how *Wolbachia* affects mosquito traits relevant to development (e.g., growth rate, immune function).
- Line 90: This section on *Wolbachia*’s antiviral properties and insecticide resistance needs to be streamlined or moved to the discussion –the sections opens with antiviral properties of *Wolbachia* but jumps to life history traits culex.
- Consistency –Cytoplasmic incompatibility to CI after first mention –throughout the document, Aedes aegypti –Ae. Aegypti and culex quinquefaciatus –cx quinquefaciatus
- Add a clear hypothesis statement at the end of the introduction-- It is unclear whether the authors expect *Wolbachia* alone to support mosquito development or if they predict extracellular microbes are essential.

**Results:**

- The results describe sterilization difficulties in *Culex quinquefasciatus* egg rafts, but the reason for poor hatching after raft disintegration is unclear—delicate wash, did the eggs sink? why is the hatch-rate poor?
- The abbreviations NS, SI and SR are not clearly eleaborated-- abbreviations should be expanded in figure legends as well as in the first mention in the main text.
- Figure 1F is empty! -- the graph is missing in the provided figure PDF—only the legend ("Dev" and "Non Dev") is present.
- Line 172-174 need to be rephrased --… monitored development daily and found no significant differences in development success rates or timing between larvae monocolonized with *E. coli* wild-type and those on AUX--- a contrast to Larval development success to pupa was slightly higher on AUX (Figure 2B-E).

**Discussion**

- The study finds that *E. coli* alone can rescue larval development, but why this occurs is unclear—I suggest that the authors provide insights into whether *E. coli* contributes specific nutrients beyond vitamin B supply.
- It is not clear whether other microbiota components (e.g., fungi or archaea) were also removed in sterilization and if this could impact the interpretation of *E. coli*’s role—this needs to be in the discussion
