# Supplementary material for: Extracellular microbes are required for mosquito development even in the presence of Wolbachia
Source: PLoS Negl Trop Dis. 2025 Sep 5;19(9):e0013481. doi: 10.1371/journal.pntd.0013481 (PMC13252830; doi:10.1371/journal.pntd.0013481)
Supplement: Attachment — Submitted filename: Rebuttal Letter.docx [file pntd.0013481.s005.docx]

Point-by-point response to the reviewers and adjustments to journal requirements

**Response to Journal Requirements:**

1. All instances of “Tetramin™" have been removed (“baby fish food”).

2. All figures have been converted to *.tif format and uploaded to the Plos server.

3. Supporting Information Legends: We have added legends for the Supporting Information files. The supplemental table summarizes statistical tests, p-values, biological replicates, and the number of larvae or mosquitoes used in each experiment.

4. The figure panel was created using BioRender.com under an academic license. We confirm compliance with PLOS’s CC BY 4.0 license.

5. Financial Disclosure updated to:

This study was funded by ANR JCJC MosMi funding to M.G. (grant ANR-18-CE15-0007) and the French Government’s Investissement d’Avenir program, Laboratoire d’Excellence ‘Integrative Biology of Emerging Infectious Diseases’ (grant ANR-10-LABX-62-IBEID). The funders had no role in study design, data collection and analysis, decision to publish, or preparation of the manuscript.

**Reviewer 1.**

This study investigates the role of extracellular microbes in *Culex quinquefasciatus* larval development when *Wolbachia* is present. By creating a gnotobiotic rearing system, the researchers were able to generate *Culex* mosquitoes that have only *Wolbachia* (germ-freeWol+), allowing them to ascertain whether *Wolbachia* alone can support larval development. The study shows that *Wolbachia* alone is not enough for development and may even impose metabolic cost on larvae. These findings fit into a broader framework of *Wolbachia*-host interactions and the overall significance of extracellular microbiota in mosquito physiology. However, certain data presentation, interpretation of metabolic interactions, and figure legibility should be improved.

**Abstract:**	The abstract provides a concise overview of the study, explaining the role of *Wolbachia* in mosquito development and the significance of extracellular microbes in *Culex quinquefasciatus*. Certain aspects require clarification, and the conclusion of *Wolbachia* as a "metabolic parasite" is not adequately supported

- Line 14 - Clarify which insects rely on *Wolbachia* for nutrition to strengthen this claim

We thank the reviewer for this suggestion. We have clarified in the abstract that nutritional mutualism with *Wolbachia* is documented in filarial nematodes, bedbugs (*Cimex lectularius*), and planthoppers (*Nilaparvata lugens, Laodelphax striatellus*), where *Wolbachia* provides vitamins such as riboflavin and biotin essential for host development.

- Line 21 - Experiment shows that *E. coli* can support development but this does not mean *Culex* mosquitoes rely only on *E. coli* in natural conditions -- "which generally grows in dirty water" could be misinterpreted as suggesting that *E. coli* is the dominant bacterium in natural *Culex* habitats. I suggest -- *E. coli* can transiently support development in the absence of native microbiota, demonstrating the role of extracellular microbes in larval growth.

We agree that the original wording could be misinterpreted. We have revised the sentence in the Abstract.

*“E. coli itself can* *support its larval development; they also underscore the importance of extracellular microbes in larval growth in the absence of native microbiota” (lines 33-34).*

This change clarifies that *E. coli* is not necessarily the dominant bacterium in natural *Culex* habitats, but serves as a model to demonstrate the essential role of extracellular microbes for larval development.

- Line 25 - this statement overgeneralizes the findings –I suggest …gnotobiology studies in *Culex quinquefasciatus* and highlight how *Wolbachia* interacts with other microbiota to influence mosquito development.

Corrected to: “In summary, this study opens the way for gnotobiology research in *Culex quinquefasciatus* and highlights the intricate interactions between *Wolbachia* and other microbiota which collectively influence mosquito development.” (lines 37-40)

**Author summary:**

- Line 33: Culex quinquefasciatus – not italicised

Corrected

- Line 40: Wolbachia - not italicised

Corrected

- Line 41: incorrectly suggests that *Wolbachia* stops being a symbiont when it imposes a metabolic burden, which is not correct symbiont can be mutualistic, commensal, or parasitic.

Thank you for the suggestion, for the author summary, we emphasize the role of *Wolbachia* as vitamin provider for hosts, and we investigated if this happens too in mosquitoes, below in the text, we claimed that “While *Wolbachia* remains a symbiont across host taxa, its interaction type varies: it acts as a nutritional mutualist in nematodes by supporting host metabolism, whereas in arthropods, it often functions as a reproductive parasite. This role involves manipulating host reproduction for its own benefit despite potentially imposing metabolic costs”.

**Introduction**

- Line 69: *Wolbachi*a's reproductive manipulations (CI, male-killing, feminization while relevant, does not directly relate to mosquito or mosquito development—needs to be removed –authours should concetrate instead summarizing how *Wolbachia* affects mosquito traits relevant to development (e.g., growth rate, immune function).

We have removed references to reproductive manipulations such as cytoplasmic incompatibility (CI), male-killing, and feminization from line 69, as these do not directly impact mosquito development.

- Line 90: This section on *Wolbachia*’s antiviral properties and insecticide resistance needs to be streamlined or moved to the discussion –the sections opens with antiviral properties of *Wolbachia* but jumps to life history traits culex.

We agree that the previous structure could be confusing and detract from the main narrative. To address this:

We have streamlined the section to clearly separate the discussion of *Wolbachia’s* antiviral properties from its effects on insecticide resistance and life history traits. The main text now focuses on developmental and physiological traits relevant to *Culex*, such as larval development, survival, and metabolic costs associated with *Wolbachia* infection, as well as the interaction with insecticide resistance genes.

- Consistency –Cytoplasmic incompatibility to CI after first mention –throughout the document, Aedes aegypti –Ae. Aegypti and culex quinquefaciatus –cx quinquefaciatus

We corrected as suggested (full names are only used in titles), except for CI that we previously used to mean confidence interval in our manuscript. We decided not to use to abbreviate neither cytoplasmic incompatibility nor confidence interval.

- Add a clear hypothesis statement at the end of the introduction-- It is unclear whether the authors expect *Wolbachia* alone to support mosquito development or if they predict extracellular microbes are essential.

We were considering both hypotheses; and even in the presence of microbes, we thought that *Wolbachia* could have positive or negative roles on development. Rather than our predictions, we ended the introduction as such:

We decided to use this approach to assess the role of *Wolbachia* during development of *Cx. quinquefasciatus*, in otherwise germ-free or monocolonized conditions (lines 124-126)

**Results:**

- The results describe sterilization difficulties in *Culex quinquefasciatus* egg rafts, but the reason for poor hatching after raft disintegration is unclear—delicate wash, did the eggs sink? why is the hatch-rate poor?

Actually we get higher hatching with raft disintegration. Another limiting factor is water depth. We have clarified it in the manuscript and explain that we hypothesize that this is linked to oxygenation requirements.

- The abbreviations NS, SI and SR are not clearly eleaborated-- abbreviations should be expanded in figure legends as well as in the first mention in the main text.

We have expanded these abbreviations in the figure and/or legends.

- Figure 1F is empty! -- the graph is missing in the provided figure PDF—only the legend ("Dev" and "Non Dev") is present.

We apologize for this.

- Line 172-174 need to be rephrased --… monitored development daily and found no significant differences in development success rates or timing between larvae monocolonized with *E. coli* wild-type and those on AUX--- a contrast to Larval development success to pupa was slightly higher on AUX (Figure 2B-E).

We rephrased it to “We monitored development daily and found minor differences in development success rates or timing between larvae monocolonized with WT and those reared with the auxotrophic strain (AUX). In the AUX group, larval development success to pupa was slightly higher, while overall development to adulthood was marginally significantly lower (Figure 2B-E).“

**Discussion**

- The study finds that *E. coli* alone can rescue larval development, but why this occurs is unclear—I suggest that the authors provide insights into whether *E. coli* contributes specific nutrients beyond vitamin B supply.

Thank you for this insightful comment. wPip is not able to synthetise all the B vitamins, so we cannot discriminate whether the lack of development is due to some B vitamins or to other factors. However, we agree that B vitamins is not the only contribution of bacteria to development and added in the introduction that “Bacteria also support lipid, protein and nucleic acid metabolism in larvae” (lines 78-79).

- It is not clear whether other microbiota components (e.g., fungi or archaea) were also removed in sterilization and if this could impact the interpretation of *E. coli*’s role—this needs to be in the discussion

Thank you for this important observation. The sterilization protocol described in the manuscript was designed to remove all culturable microbiota from the mosquito eggs, including bacteria, fungi, and potentially archaea, by using sodium hypochlorite and ethanol treatments followed by incubation in sterile conditions. Sterility was confirmed by plating egg samples on Lysogeny Broth (LB) agar, which primarily detects bacteria but can also reveal the presence of some fungi.

**Reviewer #2**

**Conclusions**

There is some overstatement of the conclusions as there is not supporting data fro some of their conclusions. Specifically that at high tetracycline concentration larval development decrease is driven by toxicity.

(see below)

**Summary and General Comments**

The manuscript “Extracellular microbes are required for mosquito development even in1
the presence of Wolbachia” by Serrato-Salas et al. presents the results of a study showing that axenic Culex mosquitoes are not benefitted by intracellular Wolbachia. Overall this is a well-crafted and written manuscript. I have a few comments that the authors may want to consider before publication.

The tetracycline data in my opinion is not completely convincing. At low tet concentrations there is a benefit to the larvae along with a reduction in Wolbachia. At higher concentrations there is a larger reduction in Wolbachia, but also detrimental effects on larval development. The authors interpret this as toxic effects on the larvae, but do not present any evidence. Couldn’t this also suggest there is a threshold level at which Wolbachia is beneficial. Too many is a burden, too few is detrimental? In either case, I think some sort of data to support the tet toxicity would be much more convincing.

We agree that this was the weaker point of the study. To validate what can be attributed to *Wolbachia* and to direct impacts of the antibiotic on the host, we performed 2 experiments. Firstly, we added a sequencing of the 16S of non-sterile and germ-free^Wol+^ adults, to show that our mosquitoes carry *Wolbachia* and no other endosymbiont (98.1% reads correspond to *Wolbachia*; no *Asaia*). Secondly, we treated germ-free Ae. aegypti larvae, which are naturally *Wolbachia*-free, with different concentrations of tetracycline (Figure 4). We observe no improvement of development at low concentration and a toxic effect at 50ug/mL or lower. We describe these experiments in the text or the results, where we also formulate alternative hypotheses.

I think the authors need to discuss the data from the paper “Three species of axenic mosquito larvae recruit a shared core of bacteria in a common garden experiment” [https://doi.org/10.1128/aem.00778-23](https://urldefense.com/v3/__https:/doi.org/10.1128/aem.00778-23__;!!JFdNOqOXpB6UZW0!tRrNwrVMK_e-w2gJHVELyloXQcCZjh8v-znV82vBs-sEuMIqBYj-AZiOsz3Clzmrn-ibaAuyoNSbRXk1p5SqFzJE$). These authors report the axenic rearing of Ae. albopictus in the absence of extracellular bacteria, while also harboring Wolbachia infections. While they do not specifiocally interrogate the effects of this, the mosquitoes have a similar developmental delay as other non-wolbachia hosting species, which lends some support to the data presented here.

Line 18 (and elsewhere): This may be a slight overstatement, maybe more appropriately insufficient to support ‘normal’ larval development. It has previously been reported that mosquitoes can not be reared axenically which was subsequently shown to no be true when reared on an appropriate diet.

We consider that this comment refers to the following sentence: “Our findings demonstrate that *Wolbachia* alone was insufficient to support larval development”. Mosquitoes have been reared on an appropriate diet, in the dark; larval development takes 3 times longer, but it does ultimately allow to produce adults (Correa et al, *Nature Communications*, 2018), which is in line with other work showing metabolic roles of microbes during larval development. We did not aim to investigate whether this protocol would allow development even in the presence of *Wolbachia*, but rather to study if *Wolbachia* supports development when providing mosquitoes, hence it was more logical to use conventional diet and conditions, except for the absence of other microbes. Hence, still think that this sentence is correct.

Line 30: There is evidence that Wolbachia interacts with more than just dengue virus. Maybe expand to arboviruses in general.

In the revised manuscript, we have expanded to “*Wolbachia* also increases resistance to viral infection, via reducing RNA virus replication. When introduced in naturally *Wolbachia*-free species, some strains of *Wolbachia* can cut down transmission of dengue, chikungunya, and West Nile viruses” (lines 108-110).

Line 234-235: There should be literature data to back up your claim the Culex may harbor more diverse and abundant bacterial communities.

We addressed in “*Culex* mosquitoes typically breed in turbid, microbe-rich water (50–52), whereas *Aedes* prefer cleaner environments (53).” (lines 371-172)

Line 271: Here you imply Wolbachia clearing rather than reduction as stated above.

We mention in the discussion that we do not necessarily observe a clearance of *Wolbachia* DNA, and that multi-generational studies are needed to fully disentangle *Wolbachia*’s role”.

Line 287 on. I do not think the data presented meets the standard of demonstrating tet toxicity. I think some kind of follow up would needed to confidently conclude this.

(see above)

**Reviewer #3**

**Methods**

Antibiotic Treatment: I presume you mean to say antibiotic was added to each “larval tray” at line 358 rather than each larvae?

There was no mistake, our larvae are indeed reared individually in 24-well plates (see “sterilization protocol”).

The amount and kind of replication is not clear in the methods. What does a replicate mean? Independent biological experimental block where fresh egg were collected from the campus or technical replicate performed at the same time from the same base material?

These replicates were performed at different times. We added this information in Methods: Section “Sterilisation protocol and bacterial inoculum”:

“For each experiment, 3-5 *Culex* egg rafts were sterilised, two flasks were found to hatch on average 1,000 sterile larvae each. Larvae were distributed into 24-well plates at a density of one larva per well, with at least 15 plates per independent biological replicate to ensure statistical power.” (lines 462-465)

We also clarified the term “replicate” in statistical analyses: “For qPCR, technical duplicates were used. The term “replicate” otherwise refers to “biological replicate” throughout the manuscript.” (lines 523-525)

The authors should provide additional detail as to the age of larvae/adults when parameters were estimated. E.g. 2F-2H. What were the ages of mosquitoes, 3A and C what instar age were larvae; 3B,D-E and J what age were adults. Also how many larvae were pooled for Wolbachia density measurements?

We added this information as follows:

Section: “Larval *development monitoring and adult manipulation.”:*

“Hatching rates were quantified microscopically 24 h post-sterilisation. Survival, pupation and development success were recorded twice and daily for 14 days. After pupae transfer in sterile cups with cotton and sugar, sex was recorded by verifying the appearance of antenna fibrils, maxillary palps and abdominal apex (…) Adults were collected 3-5 days post emergence for wing size quantification and 10 days post-emergence for CFU experiments.” (lines 469-472; 476-477)

We also added more details in figure legends.

How were E. coli densities performed as well as CFU measurements?

Some details can be found in “assessment of bacterial decolonization” and “*Wolbachia* detection” and may have been missed in the former version. We also added details in the corresponding sections as follows: “For sterile larvae, pools of ≥30 first-instar larvae were processed per sample”. (lines 495-496)

**Results**

Figure 1: Define abbreviations (e.g. NS, SI, SR) for B-D. 1F is also missing data.

and

Figure 3: See comments regarding age and pool size in methods
All figures: explain n(+) and n(-) in figures

We apologize for the missing data in 1F. We removed some abbreviations in figures and defined the remaining ones in figure legends. We added the explanation for n(+) and n(-) abbreviations in the legends of Figures 2 and 3: “n(+): number of individuals harbouring 1 or more CFUs. n(-): number of individuals harbouring 0 CFU”

**Conclusions**

I question some of the conclusions about the impact of Wolbachia on development success. The assessment of larvae and mosquitoes during the same generation as tetracycline treatment provides a confounding interaction here. As the authors cite themselves, it has been shown that tetracyline treatment can have multi-generational impacts on host metabolism and mtDNA (Ballard reference). Additional studies also support negative impacts of tetracycline on mitochondrial function resulting in wide range gene misregulation and stress due from mitonuclear protein imbalance, (Moullan et al, Cell Reports 2015; Chatzispyrou et al Cancer research 2015). It is possible that the effects observed by the authors are due to the use of antibiotic alone.

It may also be possible that the mosquito harbours other endosymbionts (e.g. Asaia) which may be altered either by the antibiotic or in response to changes in gut bacteria and Wolbachia density changes. Have the authors performed any tests to confirm Wolbachia is the only endosymbiont in their material?

We agree that this was the weaker point of the study. To validate what can be attributed to *Wolbachia* and to direct impacts of the antibiotic on the host, we performed 2 experiments. Firstly, we added a sequencing of the 16S of non-sterile and germ-free^Wol+^ adults, to show that our mosquitoes carry *Wolbachia* and no other endosymbiont (98.1% reads correspond to *Wolbachia*; no *Asaia*). Secondly, we treated germ-free Ae. aegypti larvae, which are naturally *Wolbachia*-free, with different concentrations of tetracycline (Figure 4). We observe no improvement of development at low concentration and a toxic effect at 50ug/mL or lower. We describe these experiments in the text or the results, where we also formulate alternative hypotheses.

We also discussed about degradation products (anhydrotetracycline) and oxidative stress.

We believe these revisions address all concerns and significantly strengthen the manuscript. Thank you for your time and constructive feedback.
