# Supplementary material for: Extracellular microbes are required for mosquito development even in the presence of Wolbachia
Source: PLoS Negl Trop Dis. 2025 Sep 5;19(9):e0013481. doi: 10.1371/journal.pntd.0013481 (PMC13252830; doi:10.1371/journal.pntd.0013481)
Supplement: Peer review history

# Peer review: Decision Letter 0

**DOI:** 10.1371/journal.pntd.0013481.r001

**Authors:** Doug Brackney

24 Mar 2025

-->PNTD-D-25-00250

Extracellular microbes are required for mosquito development even in the presence of Wolbachia

PLOS Neglected Tropical Diseases

Dear Dr. Gendrin,

Thank you for submitting your manuscript to PLOS Neglected Tropical Diseases. After careful consideration, we feel that it has merit but does not fully meet PLOS Neglected Tropical Diseases's publication criteria as it currently stands. Therefore, we invite you to submit a revised version of the manuscript that addresses the points raised during the review process.

Please submit your revised manuscript within 60 days May 23 2025 11:59PM. If you will need more time than this to complete your revisions, please reply to this message or contact the journal office at plosntds@plos.org. When you're ready to submit your revision, log on to https://www.editorialmanager.com/pntd/ and select the 'Submissions Needing Revision' folder to locate your manuscript file.

Please include the following items when submitting your revised manuscript:

* A rebuttal letter that responds to each point raised by the editor and reviewer(s). You should upload this letter as a separate file labeled 'Response to Reviewers'. This file does not need to include responses to any formatting updates and technical items listed in the 'Journal Requirements' section below.

* A marked-up copy of your manuscript that highlights changes made to the original version. You should upload this as a separate file labeled 'Revised Manuscript with Track Changes'.

* An unmarked version of your revised paper without tracked changes. You should upload this as a separate file labeled 'Manuscript'.

If you would like to make changes to your financial disclosure, competing interests statement, or data availability statement, please make these updates within the submission form at the time of resubmission. Guidelines for resubmitting your figure files are available below the reviewer comments at the end of this letter.

We look forward to receiving your revised manuscript.

Kind regards,

Doug E Brackney, PhD

Academic Editor

PLOS Neglected Tropical Diseases

Amy Morrison

Section Editor

PLOS Neglected Tropical Diseases

Shaden Kamhawi

co-Editor-in-Chief

PLOS Neglected Tropical Diseases

orcid.org/0000-0003-4304-636XX

Paul Brindley

co-Editor-in-Chief

PLOS Neglected Tropical Diseases

orcid.org/0000-0003-1765-0002

**Journal Requirements:**

1) We do not publish any copyright or trademark symbols that usually accompany proprietary names, eg ©,  ®, or TM  (e.g. next to drug or reagent names). Therefore please remove all instances of trademark/copyright symbols throughout the text, including:

- TM on page: 12.

2) Please upload all main figures as separate Figure files in .tif or .eps format. For more information about how to convert and format your figure files please see our guidelines:

https://journals.plos.org/plosntds/s/figures

3) We have noticed that you have uploaded Supporting Information files, but you have not included a list of legends. Please add a full list of legends for your Supporting Information files after the references list.

4) Some material included in your submission may be copyrighted. According to PLOSu2019s copyright policy, authors who use figures or other material (e.g., graphics, clipart, maps) from another author or copyright holder must demonstrate or obtain permission to publish this material under the Creative Commons Attribution 4.0 International (CC BY 4.0) License used by PLOS journals. Please closely review the details of PLOSu2019s copyright requirements here: PLOS Licenses and Copyright. If you need to request permissions from a copyright holder, you may use PLOS's Copyright Content Permission form.

Please respond directly to this email and provide any known details concerning your material's license terms and permissions required for reuse, even if you have not yet obtained copyright permissions or are unsure of your material's copyright compatibility. Once you have responded and addressed all other outstanding technical requirements, you may resubmit your manuscript within Editorial Manager.

Potential Copyright Issues:

i) Figure 2A. Please confirm whether you drew the images / clip-art within the figure panels by hand. If you did not draw the images, please provide (a) a link to the source of the images or icons and their license / terms of use; or (b) written permission from the copyright holder to publish the images or icons under our CC BY 4.0 license. Alternatively, you may replace the images with open source alternatives. See these open source resources you may use to replace images / clip-art:

- https://commons.wikimedia.org

- https://openclipart.org/.

5) Please amend your detailed Financial Disclosure statement. This is published with the article. It must therefore be completed in full sentences and contain the exact wording you wish to be published.

1) State what role the funders took in the study. If the funders had no role in your study, please state: "The funders had no role in study design, data collection and analysis, decision to publish, or preparation of the manuscript."

2) If any authors received a salary from any of your funders, please state which authors and which funders..

**Reviewers' Comments:**

**Comments to the Authors:**

**Please note that one of the reviews is uploaded as an attachment.**

Reviewer's Responses to Questions

**Key Review Criteria Required for Acceptance?**

As you describe the new analyses required for acceptance, please consider the following:

**Methods**

-Are the objectives of the study clearly articulated with a clear testable hypothesis stated?

-Is the study design appropriate to address the stated objectives?

-Is the population clearly described and appropriate for the hypothesis being tested?

-Is the sample size sufficient to ensure adequate power to address the hypothesis being tested?

-Were correct statistical analysis used to support conclusions?

-Are there concerns about ethical or regulatory requirements being met?

Reviewer #1: (No Response)

Reviewer #2: The methods are appropriate and well described.

Reviewer #3: Antibiotic Treatment: I presume you mean to say antibiotic was added to each “larval tray” at line 358 rather than each larvae?

The amount and kind of replication is not clear in the methods. What does a replicate mean? Independent biological experimental block where fresh egg were collected from the campus or technical replicate performed at the same time from the same base material?

The authors should provide additional detail as to the age of larvae/adults when parameters were estimated. E.g. 2F-2H. What were the ages of mosquitoes, 3A and C what instar age were larvae; 3B,D-E and J what age were adults. Also how many larvae were pooled for Wolbachia density measurements?

How were E. coli densities performed as well as CFU measurements?

**********

**Results**

-Does the analysis presented match the analysis plan?

-Are the results clearly and completely presented?

-Are the figures (Tables, Images) of sufficient quality for clarity?

Reviewer #1: (No Response)

Reviewer #2: The results are well presented and described

Reviewer #3: Figure 1: Define abbreviations (e.g. NS, SI, SR) for B-D. 1F is also missing data.

Figure 3: See comments regarding age and pool size in methods

All figures: explain n(+) and n(-) in figures

**********

**Conclusions**

-Are the conclusions supported by the data presented?

-Are the limitations of analysis clearly described?

-Do the authors discuss how these data can be helpful to advance our understanding of the topic under study?

-Is public health relevance addressed?

Reviewer #1: (No Response)

Reviewer #2: There is some overstatement of the conclusions as there is not supporting data fro some of their conclusions. Specifically that at high tetracycline concentration larval development decrease is driven by toxicity.

Reviewer #3: I question some of the conclusions about the impact of Wolbachia on development success. The assessment of larvae and mosquitoes during the same generation as tetracycline treatment provides a confounding interaction here. As the authors cite themselves, it has been shown that tetracyline treatment can have multi-generational impacts on host metabolism and mtDNA (Ballard reference). Additional studies also support negative impacts of tetracycline on mitochondrial function resulting in wide range gene misregulation and stress due from mitonuclear protein imbalance, (Moullan et al, Cell Reports 2015; Chatzispyrou et al Cancer research 2015). It is possible that the effects observed by the authors are due to the use of antibiotic alone.

It may also be possible that the mosquito harbours other endosymbionts (e.g. Asaia) which may be altered either by the antibiotic or in response to changes in gut bacteria and Wolbachia density changes. Have the authors performed any tests to confirm Wolbachia is the only endosymbiont in their material?

**********

**Editorial and Data Presentation Modifications?**

Use this section for editorial suggestions as well as relatively minor modifications of existing data that would enhance clarity. If the only modifications needed are minor and/or editorial, you may wish to recommend “Minor Revision” or “Accept”.

Reviewer #1: (No Response)

Reviewer #2: (No Response)

Reviewer #3: Line 63: I would alter this to emphasize that females can rescue CI. Males that cause CI can still fertilize eggs, those eggs just don’t develop correctly after the first mitotic divisions.

Line 81: I would also emphasize there are questions about the validity of those potential infections, e.g. see Ross and Hoffmann, Ecology and Evolution 2024; Chrostek and Gerth, mBio 2019.

Sentence at line 90 seems out of place.

**********

**Summary and General Comments**

Use this section to provide overall comments, discuss strengths/weaknesses of the study, novelty, significance, general execution and scholarship. You may also include additional comments for the author, including concerns about dual publication, research ethics, or publication ethics. If requesting major revision, please articulate the new experiments that are needed.

Reviewer #1: (No Response)

Reviewer #2: The manuscript “Extracellular microbes are required for mosquito development even in1

the presence of Wolbachia” by Serrato-Salas et al. presents the results of a study showing that axenic Culex mosquitoes are not benefitted by intracellular Wolbachia. Overall this is a well-crafted and written manuscript. I have a few comments that the authors may want to consider before publication.

The tetracycline data in my opinion is not completely convincing. At low tet concentrations there is a benefit to the larvae along with a reduction in Wolbachia. At higher concentrations there is a larger reduction in Wolbachia, but also detrimental effects on larval development. The authors interpret this as toxic effects on the larvae, but do not present any evidence. Couldn’t this also suggest there is a threshold level at which Wolbachia is beneficial. Too many is a burden, too few is detrimental? In either case, I think some sort of data to support the tet toxicity would be much more convincing.

I think the authors need to discuss the data from the paper “Three species of axenic mosquito larvae recruit a shared core of bacteria in a common garden experiment” https://doi.org/10.1128/aem.00778-23. These authors report the axenic rearing of Ae. albopictus in the absence of extracellular bacteria, while also harboring Wolbachia infections. While they do not specifiocally interrogate the effects of this, the mosquitoes have a similar developmental delay as other non-wolbachia hosting species, which lends some support to the data presented here.

Line 18 (and elsewhere): This may be a slight overstatement, maybe more appropriately insufficient to support ‘normal’ larval development. It has previously been reported that mosquitoes can not be reared axenically which was subsequently shown to no be true when reared on an appropriate diet.

Line 30: There is evidence that Wolbachia interacts with more than just dengue virus. Maybe expand to arboviruses in general.

Line 234-235: There should be literature data to back up your claim the Culex may harbor more diverse and abundant bacterial communities.

Line 271: Here you imply Wolbachia clearing rather than reduction as stated above.

Line 287 on. I do not think the data presented meets the standard of demonstrating tet toxicity. I think some kind of follow up would needed to confidently conclude this.

Reviewer #3: Serrato-Sales et al. develop a protocol to generate germ-free Culex quinquefasciatus mosquitoes that only harbour Wolbachia. They expand on work performed in Ae. aegypti, optimising their previous egg sterilization method to successfully remove gut bacteria in Culex quinquefasciatus. Their removal of gut bacteria allowed them to show that Wolbachia alone did not support larval development. The authors then attempt to modulate Wolbachia levels using tetracycline to measure any impact of the presence of Wolbachia on developmental success of germ-free mosquitoes. They show that Wolbachia presence may impact development (although see my comments below), and that tetracyline can be toxic to larvae at high levels.

The work to create germ-free mosquitoes is very strong and supported by data. However, the conclusions drawn by the authors around Wolbachia impact on development are confounded by the active treatment of tetracyline. While their method of select E. coli removal provides an innovative method to selectively target Wolbachia, as mentioned in the conclusions section, tetracyline has known effects on hosts and, therefore, these results cannot be disentangled. As such their current data to not support this final conclusion. If the authors really want to make this conclusion, they would need to completely remove Wolbachia through tetracyline treatment over multiple generations, allow the mosquitoes to recover from the treatment, and then perform similar types of comparisons.

The authors also left out quite a bit of methods from this manuscript. I presume they are published in previous work, but that also needs to be described and cited in the methods section.

**********

PLOS authors have the option to publish the peer review history of their article (what does this mean? ). If published, this will include your full peer review and any attached files.

If you choose “no”, your identity will remain anonymous but your review may still be made public.

**Do you want your identity to be public for this peer review?** For information about this choice, including consent withdrawal, please see our Privacy Policy .

Reviewer #1: **Yes:**  David Omondi

Reviewer #2: No

Reviewer #3: No

[NOTE: If reviewer comments were submitted as an attachment file, they will be attached to this email and accessible via the submission site. Please log into your account, locate the manuscript record, and check for the action link "View Attachments". If this link does not appear, there are no attachment files.]

**Figure resubmission:**

While revising your submission, please upload your figure files to the Preflight Analysis and Conversion Engine (PACE) digital diagnostic tool, https://pacev2.apexcovantage.com/. PACE helps ensure that figures meet PLOS requirements. To use PACE, you must first register as a user. Registration is free. Then, login and navigate to the UPLOAD tab, where you will find detailed instructions on how to use the tool. If you encounter any issues or have any questions when using PACE, please email PLOS at figures@plos.org. Please note that Supporting Information files do not need this step. If there are other versions of figure files still present in your submission file inventory at resubmission, please replace them with the PACE-processed versions.

**Reproducibility:**

To enhance the reproducibility of your results, we recommend that authors of applicable studies deposit laboratory protocols in protocols.io, where a protocol can be assigned its own identifier (DOI) such that it can be cited independently in the future. Additionally, PLOS ONE offers an option to publish peer-reviewed clinical study protocols. Read more information on sharing protocols at https://plos.org/protocols?utm_medium=editorial-email&utm_source=authorletters&utm_campaign=protocols-->

Attachment Submitted filename: Manuscript Number PNTD-D-25-00250.docx

# Author comment: Author response to Decision Letter 1

**DOI:** 10.1371/journal.pntd.0013481.r002

13 Aug 2025

Attachment Submitted filename: Rebuttal Letter.docx

# Editor report: Decision Letter 1

**DOI:** 10.1371/journal.pntd.0013481.r003

**Authors:** Doug Brackney

15 Aug 2025

Dear Dr Gendrin,

We are pleased to inform you that your manuscript 'Extracellular microbes are required for mosquito development even in the presence of Wolbachia' has been provisionally accepted for publication in PLOS Neglected Tropical Diseases.

Before your manuscript can be formally accepted you will need to complete some formatting changes, which you will receive in a follow up email. A member of our team will be in touch with a set of requests.

Please note that your manuscript will not be scheduled for publication until you have made the required changes, so a swift response is appreciated.

IMPORTANT: The editorial review process is now complete. PLOS will only permit corrections to spelling, formatting or significant scientific errors from this point onwards. Requests for major changes, or any which affect the scientific understanding of your work, will cause delays to the publication date of your manuscript.

Should you, your institution's press office or the journal office choose to press release your paper, you will automatically be opted out of early publication. We ask that you notify us now if you or your institution is planning to press release the article. All press must be co-ordinated with PLOS.

Thank you again for supporting Open Access publishing; we are looking forward to publishing your work in PLOS Neglected Tropical Diseases.

Best regards,

Doug E Brackney, PhD

Academic Editor

PLOS Neglected Tropical Diseases

Amy Morrison

Section Editor

PLOS Neglected Tropical Diseases

Shaden Kamhawi

co-Editor-in-Chief

PLOS Neglected Tropical Diseases

orcid.org/0000-0003-4304-636XX

Paul Brindley

co-Editor-in-Chief

PLOS Neglected Tropical Diseases

orcid.org/0000-0003-1765-0002

***********************************************************

p.p1 {margin: 0.0px 0.0px 0.0px 0.0px; line-height: 16.0px; font: 14.0px Arial; color: #323333; -webkit-text-stroke: #323333}span.s1 {font-kerning: none-->

# Editor report: Acceptance letter

**DOI:** 10.1371/journal.pntd.0013481.r004

**Authors:** Doug Brackney

Dear Dr Gendrin,

We are delighted to inform you that your manuscript, "

Extracellular microbes are required for mosquito development even in the presence of *Wolbachia* ," has been formally accepted for publication in PLOS Neglected Tropical Diseases.

We have now passed your article onto the PLOS Production Department who will complete the rest of the publication process. All authors will receive a confirmation email upon publication.

The corresponding author will soon be receiving a typeset proof for review, to ensure errors have not been introduced during production. Please review the PDF proof of your manuscript carefully, as this is the last chance to correct any scientific or type-setting errors. Please note that major changes, or those which affect the scientific understanding of the work, will likely cause delays to the publication date of your manuscript. Note: Proofs for Front Matter articles (Editorial, Viewpoint, Symposium, Review, etc...) are generated on a different schedule and may not be made available as quickly.

Soon after your final files are uploaded, the early version of your manuscript will be published online unless you opted out of this process. The date of the early version will be your article's publication date. The final article will be published to the same URL, and all versions of the paper will be accessible to readers.

You will receive an invoice from PLOS for your publication fee after your manuscript has reached the completed accept phase. If you receive an email requesting payment before acceptance or for any other service, this may be a phishing scheme. Learn how to identify phishing emails and protect your accounts at https://explore.plos.org/phishing.

Thank you again for supporting open-access publishing; we are looking forward to publishing your work in PLOS Neglected Tropical Diseases.

Best regards,

Shaden Kamhawi

co-Editor-in-Chief

PLOS Neglected Tropical Diseases

Paul Brindley

co-Editor-in-Chief

PLOS Neglected Tropical Diseases
